# Il Secolo Omiopatico

**Published:** 1891-10

**Authors:** 


### BOOK NOTICES.

Il Secolo Omiopatico. Periodico Mensile redatto dal Dottor Giulio Palumbo, Napoli, Tipo-Lithografia Luigi Pagnotta Via Tribunali, 12 e 13. Anno I, numero 1.

This is the first number of a new monthly journal devoted to Homœopathy, and published by Dr. Palumbo, of Naples. Its name may be translated *The Homœopathic Cycle.* It has for motto a saying of Hahnemann, "Seek diligently the truth and you will find it." We wish the new journal all success.

